# Transcriptional mechanisms underlying life‐history responses to climate change in the three‐spined stickleback

**DOI:** 10.1111/eva.12487

**Published:** 2017-05-15

**Authors:** Sin‐Yeon Kim, Maria M. Costa, Anna Esteve‐Codina, Alberto Velando

**Affiliations:** ^1^ Departamento de Ecoloxía e Bioloxía Animal Universidade de Vigo Vigo Spain; ^2^ CNAG‐CRG Barcelona Institute of Science and Technology Barcelona Spain; ^3^ Universitat Pompeu Fabra Barcelona Spain

**Keywords:** climate change, gene expression, hormone, life‐history, metabolism, phenotypic plasticity, quantitative genetics, transcriptomics

## Abstract

Phenotypic plasticity, the ability of an organism to express different phenotypes depending on the environment, provides an important mechanism by which an animal population can persist under rapid climate change. We experimentally tested both life‐history and transcriptional responses of an ecological model species, the three‐spined stickleback, to warm acclimation at the southern edge of its European range. We explored cross‐environment genetic correlations of key life‐history traits in male sticklebacks exposed to long‐term temperature changes to examine whether the plasticity pattern was variable among genotypes by using a character‐state approach. We also studied gene expression plasticity by analysing both whole‐transcriptome and candidate gene expression in brain and liver. Male sticklebacks that developed under warmer conditions during winter were smaller in size and invested less in nuptial coloration at the beginning of the breeding season, showing similar responses across different genotypes. The lack of genetic variation in life‐history responses may limit any future evolution of the thermal reaction norm in the study population. After long‐term exposure to increased winter temperatures, genes responsible for several metabolic and oxidation–reduction processes were upregulated, and some hormone genes involved in growth and reproduction were downregulated in the brain. In the liver, there was no significantly represented gene ontology by the differentially expressed genes. Since a higher temperature leads to a higher resting metabolic rate, living in warmer environments may incur higher energetic costs for ectotherms to maintain cellular homoeostasis, resulting in negative consequences for life‐history traits. The expression of genes related to metabolism, cellular homoeostasis and regulatory signalling may underlie temperature‐induced changes in life history.

## 1 INTRODUCTION

There is increasing evidence that climate change is influencing biodiversity through increased extinction risk and altered geographical distribution for many species worldwide (Deutsch et al., 2008; Thomas et al., 2004). Thus, the responses of animals to climate change and associated fitness consequences have recently been the focus of ecological and evolutionary researches. The most important mechanisms by which an animal population can respond to climate change include population‐level evolutionary changes in response to thermal stress and individual‐level phenotypic plasticity (Barrett & Hendry, 2012; Bradshaw & Holzapfel, 2008; Charmantier & Gienapp, 2014). In particular, phenotypic plasticity, the ability of a genotype to produce different phenotypes when exposed to different environments (Pigliucci, 2001), allows rapid responses to climate change compared to adaptive evolution. To be effective, phenotypic plasticity should be able to produce an adaptive response to an environmental cue in an appropriate and timely manner. For example, individual adjustment of egg‐laying date in response to the prelaying temperature has enabled a bird population to attain optimal reproductive timing, producing a better phenotype–environment match (Charmantier et al., 2008). Nevertheless, increased temperatures often have negative impacts on fitness‐related traits of animals, particularly in populations at the current low‐latitude margins of species’ distribution (i.e., rear edge populations) that live near the species’ thermal limits (Somero, 2010). Rear edge populations are more vulnerable in a changing environment than those from the centre (Vucetich & Waite, 2003) because they are typically small and geographically isolated populations and tend to have reduced within‐population genetic diversity (Chang, Kim, Park, & Maunder, 2004). These populations are particularly important for studying the survival and evolutionary adaptation of species under climate change because understanding their capacity for thermal acclimation will allow us to predict the effects of future climate on the species across its range (Hampe & Petit, 2005; Razgour et al., 2013).

Different genotypes from the same population as well as different populations may vary in the level of their response to environmental conditions. This variation in plasticity can be detected and quantified by testing genotype‐by‐environment interaction (G × E). Although the optimal plasticity in which an organism produces an appropriate phenotypic response will increase its fitness, the rareness of this capacity in natural populations suggests that there are constraints on the evolution of plasticity (Murren et al., 2015). Important constraints on the evolution of adaptive plasticity are costs of plasticity, which lead to decremented fitness in a highly plastic genotype relative to a less plastic genotype (Callahan, Maughan, & Steiner, 2008; DeWitt, Sih, & Wilson, 1998; Ghalambor, McKay, Carroll, & Reznick, 2007). Fitness costs of plasticity may come in the form of maintenance of physiological machinery underlying plastic phenotypes. Here, it is important to distinguish costs of plasticity from costs of phenotype, which refers to the fitness trade‐offs due to allocation of resources to one trait versus another (Callahan et al., 2008). Although an increasing number of empirical studies have reported developmental changes in behavioural or life‐history traits in response to sustained and consistent climate change and sought to understand the adaptive significance of such changes, relatively few studies have focussed on negative aspects of plasticity despite its importance for the fate of populations in a changing environment (Merilä & Hendry, 2014).

Ecological studies of how climate change impacts animal populations are mainly focussed on ecologically important traits that directly affect population growth through their influence on life history (Chevin, Lande, & Mace, 2010). However, it is also necessary to explore evolutionary mechanisms underlying temperature‐induced phenotypic plasticity to understand costs associated with plasticity and predict population persistence under future climate change. Life‐history changes in response to climate change may be a consequence of temperature‐induced responses at the physiological or molecular levels. In vertebrates, control systems translate environmental cues perceived by sense organs into molecular (neuroendocrine) signals that induce programmed responses in gene expression and physiology during development (Lessells, 2008). Temperature change during development has both immediate and persistent effects on thermal acclimation capacity of ectotherm animals at the cellular and molecular levels of biological organization (Komoroske, Connon, Jeffries, & Fangue, 2015; Scott & Johnston, 2012; Shama et al., 2016; Tchernov et al., 2011; Veilleux et al., 2015). The ability of an individual to perceive thermal stress and cope physiologically with the challenge should have strong influences on its homoeostasis and survival. However, the energetic costs of this ability can compromise other life‐history components such as growth, immune function and reproduction, resulting in inevitable sublethal impacts (Hoffmann, 1995). Indeed, a recent study of the three‐spined stickleback (*Gasterosteus aculeatus* Linnaeus 1758) has shown that exposure to a simulated heat wave induced a long‐lasting immune disorder (Dittmar, Janssen, Kuske, Kurtz, & Scharsack, 2014).

Recent advances in gene expression technology make it possible to study phenotypic plasticity from an integrative perspective to illustrate the cellular and molecular mechanisms underlying plasticity of ecologically important traits (Aubin‐Horth & Renn, 2009). Indeed, the impacts of both acute and long‐term thermal stress on transcriptomic responses have been explored in recent studies (reviewed in Logan & Buckley, 2015). It is well demonstrated across taxa that acute heat stress induces molecular chaperoning of macromolecules mainly by upregulating heat‐shock proteins (*Hsps*) (reviewed in Feder & Hofmann, 1999; Logan & Buckley, 2015; Tomanek, 2010). In contrast, constant long‐term exposure to warm temperatures in early life induces little or no heat‐shock response at the transcriptional level (Logan & Somero, 2010; Podrabsky & Somero, 2004) but may elicit a cellular homoeostasis response that persists to adult life (Kültz, 2005; Schulte, 2004). Some studies have shown that developmental changes in gene expression during warm acclimation involved protein biosynthesis, active ion transport, oxygen delivery and biochemical remodelling of proteins and cells, all of which are energetically costly process (Logan & Somero, 2010; Pörtner, 2004; Smith, Bernatchez, & Beheregaray, 2013).

There is a positive relationship between environmental temperature and resting metabolic rate either within individuals that experience a temperature change or among populations or species that have adapted to divergent thermal environments over evolutionary time, particularly in ectotherms whose body temperatures conform to environmental temperatures (Clarke & Fraser, 2004). It is partly because temperature directly influences kinetic energy of cellular components, demanding more energy at higher temperatures (Gillooly, Brown, West, Savage, & Charnov, 2001). On the other hand, individual proteins degrade faster at higher temperatures, and absolute rate of protein synthesis is a major contributor to basal metabolic rate. Thus, the resting metabolic rate of each species or population should represent its evolutionary optima according to the trade‐off between the energetic demands needed for its lifestyle and the somatic maintenance at its environmental temperature (Clarke, 1993, 2004). When animals are exposed to warmer temperatures than the temperature at which the population or species has been evolutionarily adapted to live, both direct and indirect effects of temperature on resting metabolic rate may induce phenotypic changes from the molecular to organismal level. The energetic costs of accelerating metabolic rate may have negative impacts on fitness‐related life‐history phenotypes. A recent study of marine sticklebacks has demonstrated that the transgenerational effects of constant acclimation of mothers to elevated temperatures lead to higher and more efficient mitochondrial respiratory capacity and small body size of offspring (Shama et al., 2016).

In the present study, we experimentally tested how long‐term exposure of young individuals to warmer temperatures during winter influences their life‐history traits during the reproductive phase using an ecological model organism, the three‐spined stickleback, at the southern edge of its European range (for studies of central and northern populations, see Leder et al., 2015; Shama et al., 2016). Although most studies of climate change impacts focus on the effects of summer temperatures, warm winters should have greater impacts on development and growth of animals with long‐lasting consequences (Williams, Henry, & Sinclair, 2015). We focussed only on male phenotypes to study molecular mechanisms underlying temperature‐induced plasticity, which can differ substantially between the sexes. In the three‐spined stickleback, the reproductive success of males depends on their investment in red nuptial coloration (Candolin, 1999). This sexual signal is costly because their maintenance requires a continual intake of carotenoids, which have other important competing functions related to body maintenance (Blount, Metcalfe, Birkhead, & Surai, 2003; Pike, Blount, Bjerkeng, Lindstrom, & Metcalfe, 2007). Most studies of warm acclimation have examined molecular processes only in some metabolically important tissues such as muscle and liver (e.g., Shama et al., 2016; Veilleux et al., 2015). We examined gene expression plasticity in brain tissue as well as liver to identify potential genes involved in the ability to perceive thermal stress and physiologically acclimate to warm temperatures.

Here, genetically related individuals originating from a wild stickleback population were reared in two environments that differed in thermal conditions (control vs. warm winter) prior to the reproductive season. We first explored G × E (i.e., variation in phenotypic responses of genotypes to different environments) of key life‐history traits in male sticklebacks exposed to long‐term temperature changes during winter by using a character‐state quantitative genetic approach (Merilä & Hendry, 2014). By testing and estimating genetic correlations between the control and warm environments, representing the presence of G × E, we tested whether the level of phenotypic plasticity is variable among genotypes in this population. We expect the absence of such variation only if there is a single, potentially optimal plasticity pattern (i.e., optimal plasticity hypothesis), which has evolved as a fixed strategy in this population because the fitness benefits of plasticity exceed any cost of plasticity. Second, we studied gene expression plasticity during warm acclimation by analysing both whole‐transcriptome and candidate gene expression (Logan & Buckley, 2015). Based on widespread thermal reaction norm for metabolic rate phenotypes (i.e., temperature dependence of metabolism hypothesis), we expected that genes related to anaerobic respiration or oxidative stress would be upregulated in the warm‐treated sticklebacks compared to the control fishes reared in normal winter conditions. Moreover, the experimental fishes were expected to invest less in fitness‐related life‐history traits (e.g., smaller body size at maturation, reduced reproductive investment and shorter lifespan) if the molecular responses to increased temperature are energetically costly (costs of phenotype).

## 2 MATERIALS AND METHODS

### 2.1 Study population, experimental treatment and life history of males

Breeding design and rearing condition are fully described in a previous study (Kim & Velando, 2015). Three‐spined sticklebacks were captured as breeding stock in the River Ulla (Galicia, Spain) in February 2013. A total of 32 full‐sibling F1 families were obtained by crossing the wild sticklebacks (16 sires and 16 dams) during April–May. Each parent fish bred twice with two different mates in two independent spawning events. Thus, each F1 fish had maternal and paternal half‐siblings as well as full‐siblings. This breeding design allowed us to estimate narrow‐sense quantitative genetic parameters by generating half‐siblings with different (clutch‐specific) maternal and common environmental effects. The stickleback populations in Galicia are at the southern edge of the species’ European range, which extends from the Iberian peninsula and the Black Sea to the north coasts of Scotland, Ireland, Norway and up to Iceland (Makinen, Cano, & Merila, 2006; Münzing, 1963). In this annual population, sticklebacks reproduce more frequently throughout a single relatively long breeding season (late February–August) compared to more northern populations (Fernández et al., 2006; Kim, Metcalfe, & Velando, 2016). Fry in each full‐sib family were reared in two or four growth tanks of 8 L (*n *= 114 tanks), each initially housing 11 or 12 full‐siblings. Two fish per tank were subsequently removed at age 5 months for another experiment (Kim, 2016). The natural seasonal photoperiod and temperature at the sampling site of the parent fish were simulated in the growth tanks by programmed illumination and flow‐through water cooling system (length of day: a maximum of 15 hr in June and a minimum of 9 hr in December; temperature: a maximum of 20°C in August and a minimum of 9°C in January). Juveniles from the 32 full‐sib families that survived until age 6 months were weighed and permanently marked with colour elastomer tags (Northwest Marine Technologies, Shaw Island, WA, USA) under a low dose of benzocaine anaesthetic to allow tracking of individual‐specific life histories (*n *= 1,038 individuals). Randomly selected fishes from the stock were sacrificed with an overdose of benzocaine anaesthetic prior to the manipulation of temperatures (*n *= 87 individuals of unknown sex) for a parallel study.

The experimental manipulation of temperatures began in November 2013 when juvenile fish were about 6–7 months old (for details, see Kim et al., 2016). Half of the growth tanks were assigned to the control and the other half to the warm winter treatment to equally allocate individuals from the same family between the two treatments. Body mass of the juvenile fish did not differ between the two treatment groups prior to the temperature manipulation (ln‐transformed body mass at age 6 months; two‐tailed *t* test: *t*_1036_ **= 1.293, *p *= .196). Water temperature in the warm‐treated tanks was maintained at 14°C during winter, whereas the temperature in the control tanks was gradually reduced from 14°C in November to 9°C in January then increased to 14°C in March, simulating the natural seasonal pattern at the sampling site of the parent fish (Kim et al., 2016). Some global warming scenarios predict an increase of 4–6°C in average winter water temperature in this latitude (Majone, Bovolo, Bellin, Blenkinsop, & Fowler, 2012). A total of 19 of 471 control fishes and 29 of 480 warm‐treated fishes died during the winter, and the mortality rate did not differ between the two treatment groups (χ^2^ **= 2.00, *p *= .157).

At the beginning of the breeding season (late February 2014), all fish were measured (standard length) in three consecutive days and the sex of individuals was determined by male sexual coloration (*n *= 474 males; *n *= 429 females). In this population, all males express red throat colour at this time of the year, although the full colouration on the lateral body surface appears later in the season in most males. All fishes were monitored daily to record the survival. The survival of males for a year from the beginning of the 2014 breeding season was analysed to test the effect of the temperature treatment by using a Cox proportional hazard model as follows: λ(*t*)* *= λ_0_(*t*)∙e^*T+f*^, where λ(*t*) is time‐dependent mortality rate*,* λ_0_ is a baseline hazard, *T* is the fixed effect of treatment and *f* is the random effect of family. The area of red ornament coloration was measured in a randomly selected subsample of male breeders (104 controls and 105 warm‐treated males, one or two males per each growth tank; for details, see Kim et al., 2016). The selected males were allocated into individual tanks containing nesting materials and shown a gravid female for 5 min twice a week to prompt expression of nuptial colour. They were photographed on their lateral side every 2 weeks during the 6 months of breeding season. The area of red ornament coloration (hue: 1–60 and 340–359; saturation: 50–255; intensity: 0–255) was determined from the digital image, and relative size of the red area was calculated as a percentage of the total lateral body area. Relative size of red area (i.e., standardized size of red area by size of the fish) in the early territorial period (i.e., 2 weeks after the allocation into an individual tank) and individual peak coloration (i.e., individual maximum colouration during the breeding season) were used in this study.

### 2.2 Quantitative genetic basis of phenotypic plasticity

Additive genetic variance and genotype‐by‐environment (temperature) interactions (G × E) in key life‐history traits of male sticklebacks (i.e., size at maturation, relative size of nuptial colour area in the early territorial period, and seasonal peak nuptial colouration) were tested using restricted maximum‐likelihood mixed models implemented in ASReml (version 3). We first estimated additive genetic variances in standard length and early and peak colorations in univariate animal models, including the overall trait mean (μ) and experimental treatment (control or warm winter scheme) as fixed effects and the additive genetic effect (*a*) based on the pedigree information (here, full‐sib and half‐sib relationships) and residual error (ɛ) as random effects. Growth tank‐specific common environment effect was initially included as an additional random effect, but it was removed from the models presented here because its variance was zero in all traits (*V*_C_ **= 0; likelihood ratio test (LRT), *p *= 1). Thus, in each univariate model a trait (*t*) of an individual *i* was specified as: *t*_*i*_ **= μ* + *treatment* + a*_*i*_ *+ *ɛ_*i*_. Total phenotypic variance was calculated as the sum of additive genetic and residual variance components (*V*_P_ **= *V*_A_ *+ V*_R_), and then heritability was calculated as the proportion of additive genetic variance in the total phenotypic variance (*h* ^2^ **= *V*_A_/*V*_P_). The significance of a fixed effect was assessed using a conditional *F* test. The significance of additive genetic variance was assessed by a LRT based on −2 × difference in log‐likelihood between the unconstrained model and a model with the variance component constrained to zero, which has one degree of freedom.

To test whether the degree of phenotypic plasticity in response to temperature treatment varied across genotypes (G × E) by testing and estimating genetic correlations between the two thermal environments, we fit bivariate animal models to treatment group‐specific traits (see Kruuk, 2004; Husby et al., 2010; Wilson et al., 2010; Kim, Noguera, Tato, & Velando, 2013; Shama, Strobel, Mark, Wegner, & Marshall, 2014 for more information about this approach). The same trait (*t*) of the two treatment groups was used in a bivariate model; for example, standard length of control and warm‐treated males was fitted as if they were two different traits: *t*_(control)*i*_ *t*_(warm)*i*_ **= μ* + a*_*i*_ *+ *ɛ_*i*_. This model decomposes a bivariate phenotypic matrix into an additive genetic matrix (*a*_*i*_) and residual (ɛ_i_) matrix in both environments. The bidimensional space of the model allows the estimation of group‐specific variances as well as the between‐group covariances. Because no individual belonged to both treatment groups (i.e., no single individual expressed a trait in both environments), residual covariance between the two groups was set to zero (Cov_R_ **= 0). Additive genetic covariances and correlations between the control and warm‐treated males were estimated based on the pedigree included as a random effect in the bivariate models. The significance of additive genetic covariance was tested by LRT between the above model (i.e., with unconstrained genetic covariance) and a model with the covariance set to zero. To test for G × E, the unconstrained model was compared to a model in which genetic correlation between environments (control and warm winter) was fixed to one by LRT. This tests the hypothesis that the genetic correlation across environments is one (*r*_G_ **= 1); that is, different genotypes show the same level of plasticity (fixed plasticity) in response to winter temperature.

### 2.3 Sampling and RNA isolation

Randomly selected subsamples (18 control and 15 warm‐acclimated males) were sacrificed by an overdose of benzocaine anaesthetic in early February 2014 when the temperature difference between the control and warm winter groups was the maximum (9 and 14°C, respectively). The whole brain and liver from the 33 males were collected using nuclease free dissection instruments. Samples were embedded in 200 μl of RiboZol (Amresco) and tissues were homogenized using RNase‐free pellet pestles (Sigma‐Aldrich) until they were totally disaggregated. A volume of 800 μl of RiboZol was added to each homogenized sample to obtain a final volume of 1 ml per sample. The prepared samples were kept at −80°C until the isolation of RNA to ensure the RNA integrity. Total RNA was isolated following the RiboZol manufacturer's instructions. All samples were treated with DNase I to remove any contaminating DNA and the treated RNA was purified using the DNA‐Free RNA kit (Zymo Research).

Among the prepared RNA samples, brain and liver samples of six individuals from three different full‐sib families (a pair of experimental and control individuals per each family, thus three individuals per treatment) were haphazardly selected for transcriptome sequencing. The quantity and quality of the selected RNA samples were assessed using an Agilent 2100 Bioanalyzer (Agilent Technologies) based on the values of RNA concentration, RNA integrity number (RIN) and rRNA ratio [28s/18s]. A total of 2 μg RNA from the selected samples were shipped to the Centre Nacional de Anàlisi Genòmica (CNAG; Barcelona; http://www.cnag.cat/) for Illumina sequencing (see below).

For real‐time qPCR assays of candidate genes, the concentration of all the RNA samples from the 33 male sticklebacks was quantified using a Synergy HT (BioTek). Only 18 of the brain RNA samples were used for the analysis of candidate gene expression because the RNA concentration was not sufficiently high in the rest. First‐strand cDNAs were synthesized with qScript cDNA Synthesis Kit (Quanta Biosciences) using 500 ng of total RNA. The cDNA was stored at −20°C until qPCR analysis of candidate genes to validate the results of RNA‐seq (whole‐transcriptome analyses).

### 2.4 Whole‐transcriptome analyses

cDNA libraries were constructed and sequenced using the Illumina (Solexa) pyrosequencing technology at the CNAG. Total RNA was assayed for quantity and quality using Qubit^®^ RNA HS Assay (Life Technologies) and RNA 6000 Nano Assay on a Bioanalyzer 2100. From total RNA, the RNASeq libraries were prepared using the TruSeq^™^ RNA Sample Prep Kit v2 (Illumina, Inc.), following the manufacturer's instructions with minor modifications. Briefly, 0.5 μg of total RNA was used as the input material for poly‐A based mRNA enrichment with oligo‐dT magnetic beads. After fragmentation (mRNA fragment size was 80–250nt, with the major peak at 130nt), the first‐ and second‐strand cDNAs were synthesized. The double‐stranded cDNA was end‐repaired, 3′adenylated and the 3′‐”T” nucleotide of the adapter was used for the Illumina indexed adapters ligation. The ligation product was enriched by 10 cycles of PCR.

Each library was sequenced using TruSeq SBS Kit v3‐HS (Illumina Inc.) in paired‐end mode with a read length of 2 × 76 bp. On average, 40 million templates were generated in a fraction of a sequencing lane on HiSeq2000. Image analysis, base calling and quality scoring of the run were processed using the manufacturer's software Real Time Analysis (RTA 1.13.48, HCS 1.5.15.1) and followed by generation of FASTQ sequence files by CASAVA. RNA‐seq reads were aligned with the GEMtools RNA‐seq pipeline v1.7 (http://gemtools.github.io), which is based on the GEM mapper (Marco‐Sola, Sammeth, Guigo, & Ribeca, 2012). The mapping is exhaustive, which means that the mapper searches for all possible mappings up to a certain number of mismatches. The pipeline aligned the reads in a sample through three phases, mapping against the *G. aculeatus* reference genome (ENSEMBL, release 82), against a reference transcriptome and against a de novo transcriptome, which was generated from the input data to detect new junction sites. By default, the mapping parameters were set as: “mismatch”* *= 0.06, “maximum edit distance”* *= 0.2 and “quality threshold”* *= 26. The transcriptome was generated using the *G. aculeatus* annotation. After mapping, all alignments were filtered to increase the number of uniquely mapped reads. The filter criteria contained a minimum intron length of 20 bp, a maximum exon overlap of 5 bp and a filter step against a reference annotation checking for consistent pairs and junctions were both sites align to the same annotated gene. Mapping statistics and expression quantification at the gene level were calculated by the GEMtools “gtfcount” tool. To normalize gene expression and identify differently expressed (DE) genes between the control and warm‐treated male sticklebacks, we fit a negative binomial generalized linear model for each tissue using the DESeq2 package (Love, Huber, & Anders, 2014) in R v.3.2.2. The normalized quantity *q*_*ij*_ was estimated based on a negative binomial distribution proportional to the concentration of cDNA fragments from the gene *i* in the sample *j* and scaled by a normalization factor accounting for differences in sequencing depth between samples. A general linear model was fitted with a logarithmic link as: log_2_(*q*_*ij*_)* *= *x*_*j*_.β_*j*_ *,* where *x*_*j*_ represents the comparison between the warm and control samples and the coefficients β_*j*_ give the log2 fold changes for gene *i* (Love et al., 2014). We performed Gene Ontology (GO) and enrichment analyses using the g:GOSt tool from g:Profiler (Reimand et al., 2016) to interpret the DE gene list. Only the expressed genes (nonzero total read) in each tissue were used as the background gene set in each enrichment analysis to avoid sampling bias (Timmons, Szkop, & Gallagher, 2015).

### 2.5 Candidate gene expression

In order to validate the results from the transcriptome analysis, the expression profiles of some candidate genes were estimated based on relative quantitation of mRNA transcripts and assayed by real‐time qPCR using a StepOnePlus Real‐Time PCR Systems (Applied Biosystems, Forest City, CA). We used the previously prepared cDNA samples of liver (*n *= 33) and brain (*n *= 18) tissues (see Section 2.3). Six DE genes from the brain transcriptome (NADH dehydrogenase subunit 5 (*ND5*); ATP synthase F0 subunit 6 (*ATP6*); somatolactin beta (*smtlb*); proopiomelanocortin a (*pomca*); thyroid‐stimulating hormone beta subunit a (*tshba*); lysine (K)‐specific demethylase 7Aa (*kdma7aa*)) and five DE genes from the liver transcriptome (apolipoprotein Ba (*apoba*); tetraspanin 13a (*tspan13a*); cat eye syndrome chromosome region, candidate 5 (*cecr5*); ATP‐binding cassette, subfamily A member 2 (*abca2*); transducin‐like enhancer of split 3a (*tle3a*)) were selected as candidate genes (see Section 3).

Gene‐specific primers were designed based on sequence information from the three‐spined stickleback genome assembly (ensembl v82; www.ensembl.org/Gasterosteus_acuelatus/). Most similar sequences were searched using Basic Local Assembly Search Tool to confirm the annotation of the sequences. Primers were synthesized by Sigma‐Aldrich Quimica (Madrid, Spain). The efficiency of gene‐specific primers was checked to ensure similar values in amplification. Details on primer sequences, amplicon sizes, melting temperatures (Tm) and efficiency are provided in Table S1. Efficiency of each amplicon was estimated from the slopes of the amplification curves for each qPCR and averaged for each gene using LinRegPCR software (Robledo et al., 2014; Ruijter et al., 2009). Selected primer was considered acceptable when the estimated efficiency was between 1.8 and 2.0 and the melting curve presented one single peak corresponding to the denaturation of a single PCR product. Elongation factor 1 alpha gene (*EF1a*) was used as a reference gene. *EF1a* has been identified as a stable housekeeping gene in a previous study of the three‐spined stickleback (Orczewska, Hartleben, & O'Brien, 2010), and our transcriptome results from this study also showed that this gene was not differentially expressed between the experimental and control males in both brain and liver tissues (all, adj‐*p *> .991). The level of expression was measured in a 20 μl reaction volume, containing 0.6 μl of each primer (10 μM), 10 μl of Luminaris Color HiGreen qPCR Master Mix, high ROX (Thermo Fisher Scientific) and 1 μl of cDNA (2 μl in the case of *smtlb* due to the low expression level). The cycling condition was set to 95°C for 10 min, followed by 40 cycles of 95°C for 15 s and 60°C for 1 min. All reactions were performed in duplicate. We analysed relative gene expression using generalized linear mixed models with a Poisson‐lognormal distribution using MCMC.qpcr package (Matz, Wright, & Scott, 2013) implemented in R v.3.2.2. Briefly, Cqs of each well were transformed into molecule counts taking the efficiency of each amplicon into account and analysed in a multivariate “informed” model, assuming *EF1a* as a stable reference gene. An important advantage of mixed modelling in the analysis of mRNA transcripts is incorporating important sources of variation as random effects in the model (Matz et al., 2013). Thus, variation in quality and quantity of biological material among samples and inter‐run variation were taken into account in the model by including sample and plate as random effects. Family identity was also included as an additional random effect. In each model, we fitted the Poisson‐lognormal rate of the transcript count for gene *g* under treatment *i*, family *j*, plate k and sample *n* as: ψ_*gijkn*_ **= *I*_*g*_ *+ B*_*ig*_ *+ t*_*n*_ *+ a*_*jg*_+ *v*_*kg*_ *+ s*_*ng*_. *I*_*g*_ is the gene‐specific intercept and accounts for different levels of expression between genes. *B*_*ig*_ is the fixed effect of treatment *i* on gene *g,* that is gene‐specific effects of experimental treatments. Gene‐specific random effects include biological replicate *n* (*t*_*n*_), accounting for the variation in quantity of biological material among samples, family *j* (*a*_*jg*_), plate k (*v*_*kg*_) and residual variation in the biological replicate *n* (*s*_*ng*_), that is the unexplained variation due to the differences between technical replicates.

## 3 RESULTS

### 3.1 Plasticity in life‐history traits and the quantitative genetic basis

**Figure 1:**
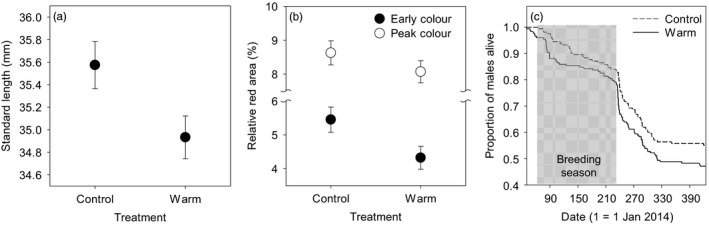
Key life‐history phenotypes of male sticklebacks with respect to the winter temperature treatment. (a) Size of fish (standard length) at the onset of the breeding season (*n *= 474 males from 32 full‐sib families, 7–23 males per family), (b) relative size of red nuptial colour area in the early territorial period and individual peak (*n *= 209 males, 3–9 males per family) and (c) survival curves from the beginning of the breeding season to the end of second winter

Male sticklebacks that experienced warm winter temperatures were significantly smaller than the control individuals at the beginning of the breeding season (Figure 1a; Table 1). Standard length showed significant heritable variance, and there was a strong positive genetic correlation across environments (i.e., control and warm winter; Table 1). This cross‐environment genetic correlation significantly differed from 0 and did not differ from 1, indicating that different genotypes showed a fixed plasticity (no G × E) in body size at sexual maturation in this stickleback population (see Fig. S1A).

**Table 1:** Quantitative genetics of life‐history traits. Additive genetic and total phenotypic variances (*V*_A_ and *V*_P_) and heritability (*h* ^2^) of fish size (standard length) at the onset of the breeding season, relative size of red nuptial colour area in the early territorial period (early colour) and individual peak colouration (peak colour), as well as the effect of experimental treatment (as a fixed effect), were analysed using univariate animal models. The significance of genetic variance was tested by likelihood ratio test (LRT). Intertreatment genetic covariances and correlations (Cov_A_ and *r*_G_) between the control and warm‐treated fishes were calculated for each trait using bivariate animal models. It was tested whether each genetic correlation differed from 0 and 1

Trait	Univariate model					
	*N*	*V*_A_ * *± *SE*	*V*_P_ ± *SE*	*h* ^2^ (95% confidence interval)	*p*	Fixed effect (treatment)
Standard length	474	2.288 ± 0.963	9.518 ± 0.711	0.240 (0.062, 0.418)	<.001	*F*_1,454.4_ * *= 4.80, *p *= .030
Early colour	209	4.715 ± 2.160	13.020 ± 1.454	0.362 (0.082, 0.642)	<.001	*F*_1,186.1_ * *= 5.39, *p *= .022
Peak colour	209	6.564 ± 2.580	11.976 ± 1.507	0.548 (0.223, 0.873)	<.001	*F*_1,184.3_ * *= 1.32, *p *= .255

	Bivariate model (cross‐environment genetic correlation)			
	*Cov*_A_ ± *SE*	*r*_G_ ± *SE*	*p* (*r*_G_ * *= 0)	*p* (*r*_G_ * *= 1)
Standard length	2.680 ± 0.994	0.998 ± 0.127	<.001	.129
Early colour	3.943 ± 2.292	0.739 ± 0.319	.047	.383
Peak colour	6.281 ± 2.580	0.984 ± 0.205	.002	.938

The control males expressed relatively larger red area, indicating a greater investment in nuptial coloration, at the beginning of their territorial phase compared to the warm‐treated males, whereas there was no difference in individual peak coloration between the treatment groups (Figure 1b; Table 1). Both early and peak coloration showed significant genetic variance, and cross‐environment genetic correlations also indicated a fixed plasticity (no G × E) in these traits (Table 1; Fig. S1B).

Analysis of survival during a year since the beginning of the breeding season revealed that the control males survived better than the warm‐treated individuals (Cox proportional hazard model including family as a random effect: β* *± *SE *= 0.246* *± 0.157, χ^2^_1_ **= 3.89, *p *= .049). The survival curves suggested that this difference was mainly due to higher mortality rates of the warm‐treated males during the early breeding season and postbreeding season (Figure 1c).

### 3.2 Plasticity in brain and liver transcriptomes

**Figure 2:**
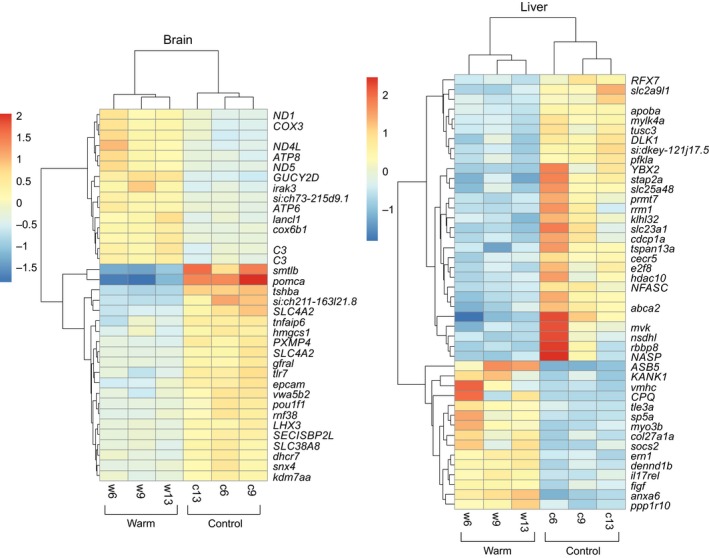
Heatmaps of the genes differentially expressed (adjusted *p *< .05) in male sticklebacks that experienced warm winter temperatures compared with control males. Separate heatmaps for brain (*n *= 36 genes) and liver (*n *= 44 genes) are shown

We used the whole‐transcriptome (RNA sequencing) data to identify genes that were differentially expressed between the male sticklebacks that experienced warm winter temperatures and the control males reared in normal winter temperatures across all annotated genes in the Ensembl stickleback genome assembly. Approximately 79 million of paired‐end reads were produced after sequencing for each sample, and 90% of them could be uniquely mapped to the reference genome. General statistics of mapping for each sample can be found in Table S2. Of 22456 genes, only those with nonzero total read count were considered for differential expression analyses (brain tissue: *n *= 20,307 genes; liver tissue: *n *= 18,781 genes). Our analyses revealed that 36 genes in the brain and 44 genes in the liver were differentially expressed between the control and warm‐treated fish at a level of adjusted *p *< .05 (Figure 2). Transcriptome results indicated a tissue‐specific regulation, and there was no similarly regulated gene between the brain and liver. There were more downregulated (i.e., lower expression in the warm‐treated fish compared with the controls) than upregulated genes in both tissues. Average log_2_ fold changes in the upregulated and downregulated genes were 0.95 and −1.20 for brain, and 1.77 and −1.80 for liver. Relative change in transcript abundance of all genes and the list of DE genes with more information are provided in Fig. S2 and Table S3.

**Figure 3:**
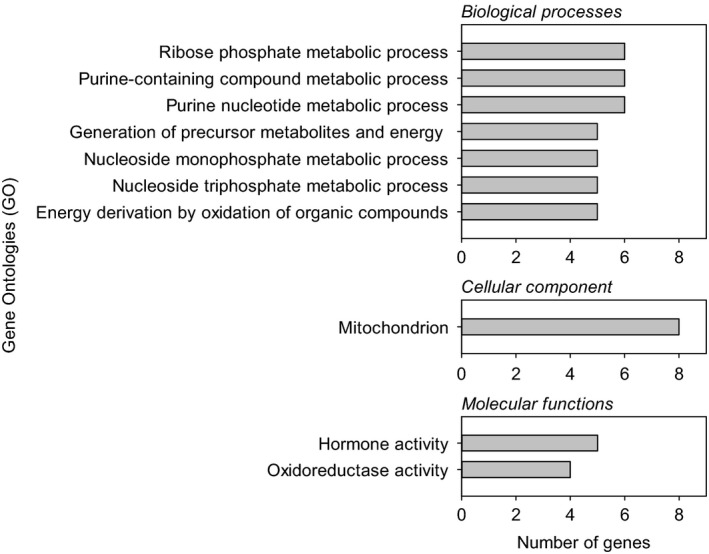
The highest‐level Gene Ontology terms (biological processes, cellular components and molecular functions) significantly represented by the differentially expressed genes in the brain of male sticklebacks that experienced warm winter temperatures

We present results from Gene Ontology (GO) and enrichment (g:GOSt) analyses using DE genes at a level of adjusted *p *< .1 (Figure 3) but analyses including only those at adjusted *p *< .05 gave similar results. In brain, the DE genes significantly represented biological process GO categories related with several metabolic processes, energy derivation by oxidation of organic compounds, and generation of precursor metabolites and energy. Mitochondrion cellular component and some molecular functions related with oxidoreductase activity and hormone activity were also significantly represented by the affected genes in brain transcriptome. Overall, these results suggested a regulation of genes related to metabolism in the brain of warm‐acclimated sticklebacks. The genes related to hormones (i.e., *smtlb*,* pomca* and *tshba*) were the most differentially expressed in the brain (log_2_ fold changes < −2, Table S3). In the liver transcriptome, there was no GO category significantly represented by the DE genes.

### 3.3 Candidate gene expression

**Figure 4:**
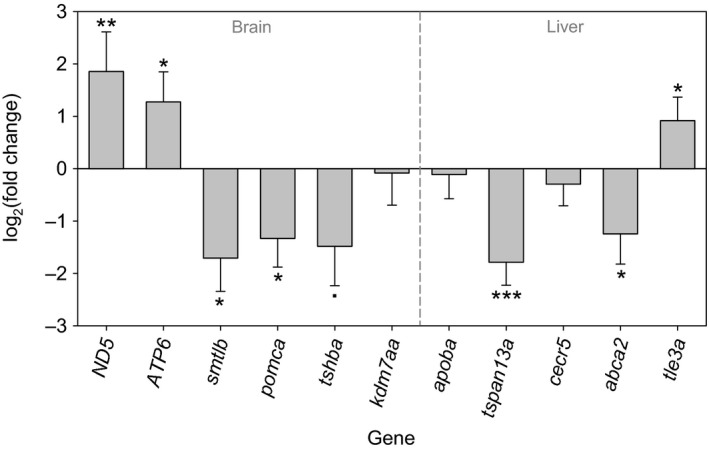
Effects of warm winter temperature treatment on the expression (via real‐time qPCR) of candidate genes in brain and liver. ***p < .001, **p < .01, *p < .05, · *p *= .05

Transcription of most candidate genes was significantly influenced by winter temperature treatment in the same pattern as the whole‐transcriptome results (Figure 4; Table S4). Genes involved in oxidation–reduction and ATP synthesis, *ND5* and *ATP6*, were upregulated in the brain tissue of the warm‐treated male sticklebacks, whereas genes related to thyroid (TH), somatolactin (SL) and proopiomelanocortin (POMC) hormones, *tshba*,* smtlb* and *pomca*, were downregulated. Another candidate gene *kdm7aa* (lysine‐specific demethylase) was downregulated in the brain, but the difference between the treatment groups was not significant. In the liver, significant downregulation of membrane protein tetraspanin gene, *tspan13a*, and ATP‐binding cassette gene, *abca2*, and upregulation of transducing‐like enhancer gene, *tle3a*, were observed. The expression of *apoba* and *cecr5* in the liver did not significantly differ between the control and experimental sticklebacks.

## 4 DISCUSSION

In this study, we simultaneously examined cross‐environment genetic correlations of ecologically important traits of male sticklebacks exposed to different temperatures and their transcriptional responses during warm acclimation to explore the genomic basis of phenotypic plasticity. Male sticklebacks that developed under warm winter conditions were smaller in size and invested less in nuptial coloration at the beginning of the reproductive phase and had lower survival compared to control individuals (see also Kim et al., 2016). Our quantitative genetic results demonstrated the presence of significant genetic variation and plasticity across different thermal environments in body size and nuptial colouration of male sticklebacks. Genetic correlations between environments showed no evidence of G × E (i.e., no genetic variation in plasticity), suggesting that different genotypes showed similar plasticity patterns for size and nuptial colour in this population. It is interesting to note that a previous study of marine sticklebacks in a more northern population (Sylt‐Rømø Bight, Germany) showed a significant G × E for size of offspring from parents acclimated to different temperatures, suggesting that reaction norms had the potential to evolve (Shama et al., 2014). The nonvariable plasticity pattern observed in our study population may be adaptive if these responses allowed the sticklebacks to avoid more catastrophic consequences of the temperature change, for example by shifting their investment from reproduction towards self‐maintenance in a warmer environment. In this low‐latitude population living close to the species’ thermal limit, this plasticity pattern may have evolved under strong natural selection, which probably eroded genetic variation in thermal reaction norms (Pigliucci, 2001). Our transcriptome results indicate that the expression of genes related to metabolism, cellular homoeostasis and regulatory signalling may underlie these changes in life‐history traits.

After long‐term exposure to increased winter temperatures, there were significant changes in the expression of some genes involved in metabolism, oxidative stress and endocrine signalling. In particular, genes responsible for several metabolic processes and oxidation–reduction were upregulated in the brain of the males reared under warm conditions. For example, genes related to mitochondrial enzyme complexes involved in the oxidative phosphorylation (e.g., ATP synthase: *ATP6*,* ATP8*; NADH dehydrogenase: *ND1*,* ND4*,* ND5*, cytochrome c oxidase: *COX3, COX6*) were upregulated. The representation of metabolic processes by the DE genes in the warm‐acclimated fishes may reflect the energetic cost of living in warm temperatures (Clarke, 2004; Clarke & Fraser, 2004). High metabolic rate induced by warm temperatures may result in oxidative stress, especially when ventilation and circulation fail to cover tissue oxygen demand (Pörtner & Farrell, 2008; Pörtner & Knust, 2007). In agreement with our results, other studies have shown that chronic thermal stress induced consistent upregulation of genes involved in protein synthesis and metabolism, as well as those for cellular homoeostasis due to increased oxidative stress, in gill or liver of other fish species (Jeffries, Hinch, Sierocinski, Pavlidis, & Miller, 2014; Komoroske et al., 2015; Shama et al., 2016; Veilleux et al., 2015; Windisch et al., 2014). In particular, it is interesting to note that the expression of genes involved in metabolism and mitochondrial protein biosynthesis matched the phenotypic patterns for mitochondrial respiratory capacity in a previous study of the three‐spined stickleback (Shama et al., 2016). These transcriptional changes during warm acclimation are probably not surprising because metabolic rate is often highly plastic in response to changing environmental conditions, including ambient temperature, in diverse taxa (Dillon, Wang, & Huey, 2010; Lovegrove, 2003; Norin, Malte, & Clark, 2016). A chronic increase in metabolic rate and oxidative stress may have negative impacts on fitness‐related traits (Burton, Killen, Armstrong, & Metcalfe, 2011; Metcalfe & Alonso‐Alvarez, 2010).

Downregulated genes in the brain transcriptome included *tshba*,* smtlb* and *pomca*, which are responsible for the secretion of TH, SL and POMC, respectively. TH is essential for larval metamorphosis in teleost fish, and its secretion is regulated by both external stimuli, such as temperature, photoperiod and olfactory cue, and internal stimuli, such as basal metabolism and growth (Blanton & Specker, 2007; Dufour & Rousseau, 2007). Since TH also regulates pelvic fin growth and pigmentation (Brown, 1997), this hormone may be required for a stickleback to begin its reproduction (Blanton & Specker, 2007). SL belongs to the growth hormone/prolactin family and regulates growth in teleost fish (Ono et al., 1990). During the period of gonadal growth, SL regulates some physiological aspect of reproduction, including the level of 11‐ketotestosterone, a fish androgen that mediates spermatogenesis, aggressive behaviour and parental behaviour (Rand‐Weaver, Swanson, Kawauchi, & Dickhoff, 1992). Melanocortins, the product of POMC gene, involve energy expenditure and sexual activity when bound to melanocortin receptors in brain (Ducrest, Keller, & Roulin, 2008). In some teleost fish, *pomca* (a paralog gene) is related to the neuroendocrine stress response and its expression is downregulated under chronic stress exposure (Wunderink et al., 2012). Taken together, the significant representation of hormone activity by some DE genes suggests that warm prereproduction conditions probably suppress growth and early reproductive investment in male sticklebacks as supported by our results of life‐history plasticity (Figure 1). These results highlight that hormones should be the focus of more studies of phenotypic plasticity because they represent the interface between the external environment and the expression of other important phenotypes (Pigliucci, 2001).

In the liver transcriptome, there was no gene ontology category clearly represented in fish reared in warmer water. Although there is a conserved transcriptome phenotype across all tissues, different tissues can show highly divergent metabolic strategies that are likely related to their physiological role within the body (Logan & Buckley, 2015). In the brain transcriptome, there were various metabolic processes represented by the DE genes. Brain is a metabolically expensive organ, and liver is the central organ that is involved in the maintenance of whole body energy homeostasis. Thus, living in warmer water may increase resting metabolic rate to different degrees in these organs as well as other energetically active tissues such as red muscle or result in a trade‐off in energy allocation among organs (Clarke & Fraser, 2004; Geluso & Hayes, 1999; Shama et al., 2016). However, we should not speculate about the metabolic strategy for liver tissue during warm acclimation based only on the transcriptomic results from fish sampled at the end of long‐term temperature manipulation. A study of zebrafish (*Danio rerio*) demonstrated that warm acclimation depleted energy stores in liver at the beginning, but then its condition recovered by physiological adjustments by the end of acclimation that lasted a month (Vergauwen, Benoot, Blust, & Knapen, 2010). In this zebrafish study, interestingly, many transcripts involved in proteolysis were downregulated in the liver after a month of warm acclimation, suggesting that energy expenditure was reduced, whereas these transcripts were upregulated after cold acclimation, indicating compensation for the decreased metabolic rate (Vergauwen et al., 2010). Although the RNA‐seq results were successfully validated for most candidate genes by real‐time qPCR, the failure in some other genes indicate that the whole‐transcriptome data from a limited number of samples should be interpreted with caution.

Accumulating evidence suggests that living in warmer temperatures than the conditions to which the species or population has adapted is energetically costly due to increased resting metabolic rate (Clarke, 2004; Gillooly et al., 2001). Resting metabolic rate is often measured and interpreted as the energy cost of self‐maintenance, which is one of the most important life‐history components together with growth and reproduction (Burton et al., 2011). Individuals must allocate limited resources among these competing functions (Stearns, 1992). The trade‐offs between self‐maintenance and other life‐history components mean that temperature‐induced changes in resting metabolic rate will probably have consequences for life‐history traits and hence fitness (Burton et al., 2011). Overall, our whole‐transcriptome and candidate gene expression results showed that warm acclimation induces genomic changes in metabolic processes and hormone actions, particularly in the brain. The compulsory changes in metabolism during warm acclimation may call for physiological changes for homoeostasis. The costs of these physiological functions may have resulted in nonvariable life‐history reaction norm, causing all genotypes and individuals to express smaller body size at maturation and reduced nuptial colouration after warm acclimation as shown in our quantitative genetic results (see also Shama et al., 2014, 2016).

A number of current studies have proposed optimistic predictions that fitness may be maintained by adaptive plasticity in animal populations experiencing sustained climate change (Barrett & Hendry, 2012; Charmantier et al., 2008; Chevin et al., 2010; Nussey, Postma, Gienapp, & Visser, 2005). However, our experiment that simultaneously explored life‐history reaction norms and genomic responses of sticklebacks at the southern edge of their distribution suggests that changes in gene expression related to metabolism, homeostasis and signalling pathways may allow animals to cope with thermal stress but result in a fitness penalty due to the costs of phenotype in warmer environments. The lack of genetic variation in life‐history responses to temperature change may constrain any further evolutionary responses to climate change in the study population. Smaller body size at maturation and reduced investment in early reproduction were fixed responses of male sticklebacks to increased winter temperatures in this population that has adapted to live in the species’ upper thermal limit over evolutionary time. This may be an optimal strategy to persist in fluctuating thermal environments, but future warming may cause local extinction of the three‐spined stickleback due to its limited genetic variation in plasticity pattern. It will be interesting to study whether this population has the capacity to shift breeding sites or timing to avoid unfavourable temperatures. Future studies need to explore more about the mechanisms of phenotypic plasticity in response to climate change at the molecular (including epigenetic), cellular and physiological levels to validate the role of phenotypic plasticity for the persistence of the species under continuing climate change across latitude.

## DATA ARCHIVING STATEMENT

All raw Illumina reads data have been deposited in the National Center for Biotechnology Information (NCBI) Sequence Read Archive (SRA): Accession SRP103174. Life‐history data, RNA‐seq count data and real‐time qPCR data used in this study are provided in Tables S5‐S7 (Supporting Information).
