# Supplementary material for: Transcriptional mechanisms underlying life‐history responses to climate change in the three‐spined stickleback
Source: Evol Appl. 2017 May 15;10(7):718–30. doi: 10.1111/eva.12487 (PMC5511362; doi:10.1111/eva.12487)
Supplement: Supplementary file 1 [file EVA-10-718-s001.pdf]

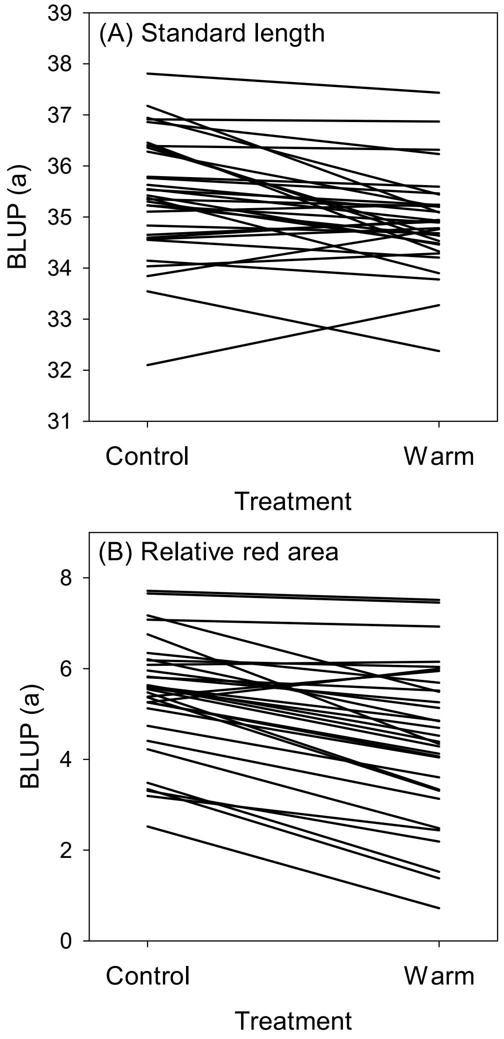

Figure S1. Reaction norm plots of (A) size of fish (standard length) at the onset of the breeding season and (B) relative size of red nuptial colour area in the early territorial period from 32 full-sib families exposed to the control and warm winter conditions. The thermal reaction norms are based on the fixed effect (temperature treatment) and the family mean best linear unbiased predictor (BLUP) values obtained from the binomial model (cross-environment genetic correlation) presented in Table 1. Thus, each line represents the predicted reaction of a single family at the additive genetic (a) level. BLUPs are used here to merely illustrate the G×E patterns, but were not used in the statistical analyses.
