# Supplementary material for: Transcriptional mechanisms underlying life‐history responses to climate change in the three‐spined stickleback
Source: Evol Appl. 2017 May 15;10(7):718–30. doi: 10.1111/eva.12487 (PMC5511362; doi:10.1111/eva.12487)
Supplement: Supplementary file 2 [file EVA-10-718-s002.pdf]

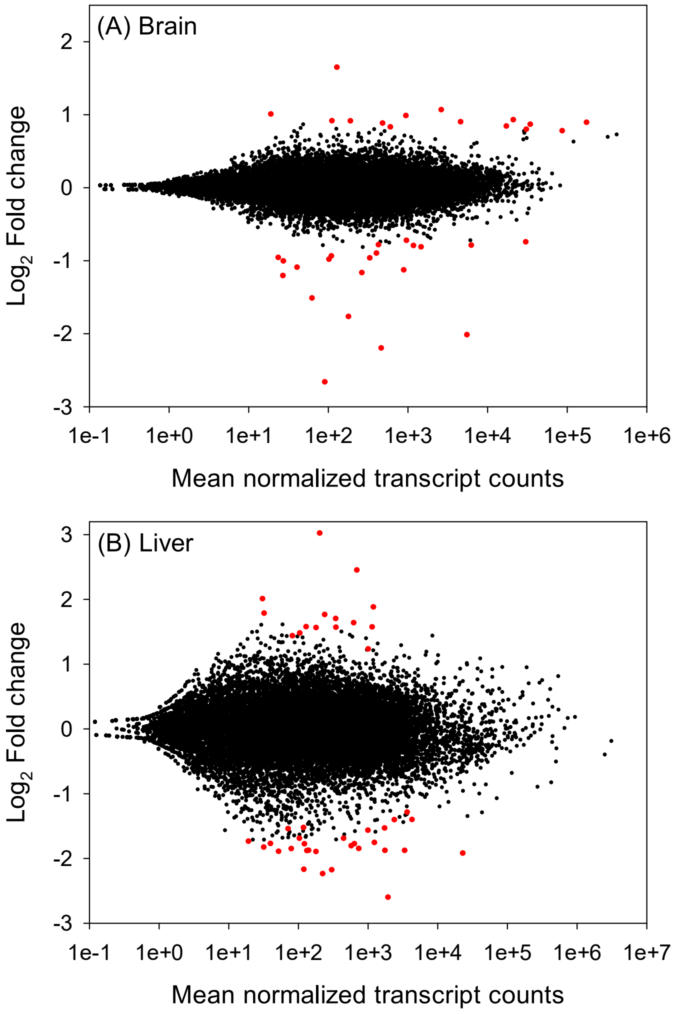

Figure S2. Relative change in transcript abundance due to increased winter temperature (14 °C *vs.* 9-14 °C) in (A) brain and (B) liver of male sticklebacks. Each data point represents the average of normalized read counts for a unique transcript. Significant DE genes (adjusted $P < 0.05$) are highlighted in red.
