# Supplementary material for: Transcriptional mechanisms underlying life‐history responses to climate change in the three‐spined stickleback
Source: Evol Appl. 2017 May 15;10(7):718–30. doi: 10.1111/eva.12487 (PMC5511362; doi:10.1111/eva.12487)
Supplement: Supplementary file 3 [file EVA-10-718-s003.pdf]

Table S1. Primers used for real-time qPCR of candidate genes.

Candidate gene	Ensembl gene ID	Forward (F) and Reverse (R) primer sequence (5′-3′)	Amplicon size (bp)	Tm (°C, F/R)	Efficiency (SE)	Repeatability
Brain						
ND5	ENSGACG00000014735	F: AGTCCTATACTCGCATCTCAACCAC<br>R:AGAGAAATCACAATCTGAGCAACAG	137	65.3/64.6	1.891 (0.003)	0.972
ATP6	ENSGACG00000020941	F: GGCTAATCTTACAGCAGGACACC<br>R: GAAGAACAAACACGTAGGCTTGA	167	64.8/64.2	1.895 (0.002)	0.967
smtlb	ENSGACT00000008758	F: CGTCTCGGAAGAATCGTGCTC<br>R: GAGCAGCCATTTGTCGGATATC	151	68.3/66.6	1.903 (0.001)	0.995
pomca	ENSGACG00000009521	F: CTATTGGTGGTTGTGGCGGTG<br>R: GACGCACTCCATCATGCTCG	111	69.4/69.4	1.903 (0.002)	0.997
tshba	ENSGACG00000005276	F: ATGGAGACTGCAGTGTTCCC<br>R: TGATCGCAACACAGTAGTCG	127	64.2/62.6	1.921 (0.002)	0.999
kdm7aa	ENSGACG00000019975	F: ATTATCAGAGATTTAAGGCGACCAG<br>R: CTTCTTCCCTGAGTTCATTCTTCAC	187	64.2/65.1	1.900 (0.002)	0.999
Liver						
apoba	ENSGACG00000009649	F: GACATCTTGGAATCTGCCACTG<br>R: CATGATGGGTTTGCTCTGCTTG	249	65.8/65.6	1.907 (0.003)	0.999
tspan13a	ENSGACG00000013330	F: GCTGAGGAAAGGAGAAACAAGC<br>R: ATTCGCTGTGTGTGTGTGTCAG	174	65.2/66.3	1.915 (0.004)	0.999
cecr5	ENSGACG00000010978	F: CCTCCAGCTTATTGTTGACGTG<br>R: ACCGATCAGAGCCTCGTACTTC	223	65.6/65.6	1.900 (0.002)	0.998
abca2	ENSGACG00000003853	F: GATCATCAACACCCTCCGTTTG<br>R: GTCCAGCCACATGAAGACAAAG	157	65.5/65.8	1.910 (0.003)	0.988
tle3a	ENSGACG00000005213	F: AATAACTCGGTATCCCCGTCTG<br>R: ACCAGGTCATCGCTTTTCTCTC	155	64.8/65.6	1.934 (0.005)	0.994
Reference						
EF1a	ENSGACT00000002834	F: CTTCTCCAACTATCCTCCCCTC<br>R: GAGAAGAGGATCCAGGGTAAGG	233	64.0/64.0	1.893 (0.005)	0.985

tshba, thyroid stimulating hormone beta a; *smtlb*, somatolactin beta; *pomca*, proopiomelanocortin a; *APT6*, mitochondrially encoded ATP synthase 6; *kdm7aa*, lysine (K)-specific demethylase 7Aa; *ND5*, NADH-ubiquinone oxidoreductase chain 5; *abca2*, ATP binding cassette A2; *apoba*, apolipoprotein Ba; *pfkla*, phosphofructokinase liver a; *cecr5*, cat eye syndrome chromosome region 5; *tspan13a*, tetraspanin 13a; *tle3a*, transducin-like enhancer of split 3a; *EF1a*, elongation factor 1 alpha
