# Supplementary material for: Transcriptional mechanisms underlying life‐history responses to climate change in the three‐spined stickleback
Source: Evol Appl. 2017 May 15;10(7):718–30. doi: 10.1111/eva.12487 (PMC5511362; doi:10.1111/eva.12487)
Supplement: Supplementary file 5 [file EVA-10-718-s005.pdf]

Table S3. Genes differentially expressed (adjusted $P < 0.05$) in brain and liver of male sticklebacks in response to increased winter temperature. Gene ID (Ensembl ID ENSGACG000000), mean normalized transcript counts, log2 fold change (FC), adjusted *P* value and description are presented for each DE gene. The gene list was sorted by adjusted *P*.

Gene ID	Gene name	Mean counts	Log_2 FC	Adj-*P*	Description
Brain					
05276	*tshba*	91.3	-2.66	0.0000	Thyroid stimulating hormone, beta, a
06593	*smtlb*	466.4	-2.20	0.0000	Somatolactin beta
09521	*pomca*	5583.4	-2.02	0.0000	Proopiomelanocortin a
08195	*si:ch211-163l21.8*	181.2	-1.77	0.0000	
01181	*GUCY2D*	129.9	1.64	0.0000	Guanylate cyclase 2D, membrane (retina-specific)
01526	*SLC4A2*	63.1	-1.52	0.0000	Solute carrier family 4 (anion exchanger), 2
05891	*PXMP4*	895.9	-1.13	0.0000	Peroxisomal membrane protein 4, 24kDa
01525	*SLC4A2*	27.2	-1.21	0.0000	Solute carrier family 4 (anion exchanger), 2
03996	*tlr7*	267.8	-1.17	0.0001	Toll-like receptor 7
08583	*cox6b1*	485.4	0.88	0.0005	Cytochrome c oxidase subunit VIb polypeptide 1
19291	*irak3*	2652.7	1.06	0.0005	Interleukin-1 receptor-associated kinase 3
18554		19.2	1.00	0.0012	
02954	*si:ch73-215d9.1*	611.0	0.83	0.0012	
06299	*gfral*	41.0	-1.09	0.0015	GDNF family receptor alpha like
06709	*pou1f1*	27.6	-1.01	0.0019	POU class 1 homeobox 1
20941	*ATP6*	87949.4	0.77	0.0068	ATP synthase F0 subunit 6 (mitochondrion)
16592	*LHX3*	23.8	-0.96	0.0082	LIM homeobox 3
20946	*ND4L*	952.7	0.98	0.0082	NADH dehydrogenase 4L
08611	*tnfaip6*	335.1	-0.97	0.0089	Tumor necrosis factor, alpha-induced protein 6
20942	*COX3*	177365.[illegible]	0.89	0.0094	Cytochrome c oxidase III
01752		21267.9	0.93	0.0094	
02632	*epcam*	103.0	-0.99	0.0094	Epithelial cell adhesion molecule
03097	*SLC38A8*	110.3	-0.94	0.0094	Solute carrier family 38, 8
20940	*ATP8*	4638.4	0.90	0.0094	ATP synthase F0 8
19373	*snx4*	6332.4	-0.79	0.0153	Sorting nexin 4
19975	*kdm7aa*	30474.7	-0.75	0.0163	Lysine (K)-specific demethylase 7Aa
19826	*C3*	191.9	0.91	0.0163	Complement component 3
15077	*vwa5b2*	406.6	-0.90	0.0188	von Willebrand factor A domain containing 5B2
16786	*SECISBP2L*	969.1	-0.73	0.0203	SECIS binding protein 2-like
14735	*lancl1*	17428.9	0.84	0.0203	LanC (bacterial lantibiotic synthetase component [illegible]
20951	*ND5*	34773.6	0.86	0.0203	NADH-ubiquinone oxidoreductase chain 5
00155	*C3*	112.4	0.91	0.0214	Complement component 3
18575	*hmgcs1*	1478.8	-0.82	0.0349	3-hydroxy-3-methylglutaryl-CoA synthase 1
20925	*ND1*	31009.4	0.80	0.0351	NADH-ubiquinone oxidoreductase chain 1
14264	*dhcr7*	431.5	-0.79	0.0369	7-dehydrocholesterol reductase
17728	*rnf38*	1187.7	-0.80	0.0492	Ring finger protein 38
Liver					
16501	*ASB5*	204.4	3.02	0.0000	Ankyrin repeat and SOCS box containing 5
18367	*anxa6*	699.3	2.45	0.0000	Annexin A6

11492		1957.7	-2.61	0.0000	
10204	*si:dkey-121j17.5*	742.7	-1.85	0.0001	
19392	*YBX2*	225.5	-2.24	0.0004	Y box binding protein 2
05421		23228.2	-1.92	0.0006	
11700	*ern1*	1156.0	1.57	0.0010	Endoplasmic reticulum to nucleus signaling 1
15332	*stap2a*	304.5	-2.18	0.0010	Signal transducing adaptor family 2a
03853	*abca2*	3405.5	-1.88	0.0013	ATP-binding cassette, sub-family A (ABC1), 2
20918	*slc23a1*	121.3	-2.17	0.0015	Solute carrier family 23 (ascorbic acid transporter), [illegible]
09292	*slc2a9l1*	141.5	-1.88	0.0055	Solute carrier family 2 (facilitated glucose [illegible]
09649	*apoba*	3675.9	-1.29	0.0056	Apolipoprotein Ba
03505	*NFASC*	1746.3	-1.54	0.0060	Neurofascin
01324	*ppp1r10*	628.8	1.63	0.0067	Protein phosphatase 1, regulatory 10
07130	*cdcp1a*	134.2	-1.88	0.0103	CUB domain containing protein 1a
05058	*sp5a*	31.0	2.00	0.0108	Sp5 transcription factor a
07908	*dennd1b*	130.4	1.57	0.0110	DENN/MADD domain containing 1B
05061	*myo3b*	347.2	1.70	0.0133	Myosin IIIB
13330	*tspan13a*	579.1	-1.81	0.0133	Tetraspanin 13a
17086	*e2f8*	52.6	-1.90	0.0133	E2F transcription factor 8
02229	*prmt7*	451.7	-1.70	0.0142	Protein arginine methyltransferase 7
07051	*CPQ*	1211.7	1.88	0.0142	Carboxypeptidase Q
09149	*mvk*	1767.3	-1.88	0.0142	Mevalonate kinase
15859	*KANK1*	241.0	1.76	0.0142	KN motif and ankyrin repeat domains 1
16856	*slc25a48*	641.9	-1.78	0.0142	Solute carrier family 25, 48
06173	*pfkla*	4313.7	-1.41	0.0148	Phosphofructokinase, liver a
16469	*mylk4a*	40.2	-1.78	0.0153	Myosin light chain kinase family, 4a
04946	*klhl32*	123.2	-1.78	0.0159	Kelch-like family member 32
02106	*rbbp8*	180.8	-1.90	0.0164	Retinoblastoma binding protein 8
09397	*DLK1*	104.5	-1.70	0.0259	Delta-like 1 homolog
16167	*NASP*	79.9	-1.86	0.0259	Nuclear autoantigenic sperm protein (histone-[illegible]
20382	*rrm1*	32.2	-1.83	0.0309	Ribonucleotide reductase M1 polypeptide
10978	*cecr5*	2406.0	-1.41	0.0326	Cat eye syndrome chromosome region, candidate 5
05213	*tle3a*	106.0	1.47	0.0331	Transducin-like enhancer of split 3a
16062	*col27a1a*	181.7	1.56	0.0331	Collagen, type XXVII, alpha 1a
18576	*tusc3*	119.7	-1.53	0.0335	Tumor suppressor candidate 3
14960	*vmhc*	32.7	1.78	0.0344	Ventricular myosin heavy chain
18285	*nsdhl*	1245.1	-1.76	0.0361	NAD(P) dependent steroid dehydrogenase-like
15838	*RFX7*	72.1	-1.55	0.0402	Regulatory factor X, 7
18998	*il17rel*	83.1	1.43	0.0460	Interleukin 17 receptor E-like
03854	*hdac10*	1007.8	-1.57	0.0492	Histone deacetylase 10
00635	*socs2*	348.8	1.56	0.0496	Suppressor of cytokine signaling 2
14402		19.5	-1.74	0.0496	
15124	*figf*	1014.5	1.23	0.0496	C-fos induced growth factor
