# Supplementary material for: Transcriptional mechanisms underlying life‐history responses to climate change in the three‐spined stickleback
Source: Evol Appl. 2017 May 15;10(7):718–30. doi: 10.1111/eva.12487 (PMC5511362; doi:10.1111/eva.12487)
Supplement: Supplementary file 6 [file EVA-10-718-s006.pdf]

Table S4. Results from MCMC GLMMs testing for the effects of warm winter treatment on candidate gene expression. Estimated coefficients and 95% confidence intervals are provided.

Gene	Estimate	Lower 95% CI	Upper 95% CI	P_{MCMC}
Brain				
ND5	1.286	0.281	2.305	0.006
ATP6	0.884	0.174	1.689	0.016
smtlb	-1.183	-2.014	-0.261	0.014
pomca	-0.924	-1.620	-0.177	0.018
tshba	-1.028	-1.927	0.079	0.054
kdm7aa	-0.0577	-0.973	0.724	0.904
Liver				
apoba	-0.0791	-0.675	0.536	0.804
tspan13a	-1.238	-1.828	-0.683	< 0.001
cecr5	-0.206	-0.795	0.329	0.450
abca2	0.862	1.629	0.071	0.034
tle3a	0.636	0.048	1.272	0.040
